# Label‐Free Imaging of Cholesterol Assemblies Reveals Hidden Nanomechanics of Breast Cancer Cells

**DOI:** 10.1002/advs.202002643

**Published:** 2020-10-08

**Authors:** Andra C. Dumitru, Danahe Mohammed, Mauriane Maja, Jinsung Yang, Sandrine Verstraeten, Aranzazu del Campo, Marie‐Paule Mingeot‐Leclercq, Donatienne Tyteca, David Alsteens

**Affiliations:** ^1^ Louvain Institute of Biomolecular Science and Technology (LIBST) Université catholique de Louvain Louvain‐la‐Neuve 1348 Belgium; ^2^ Cell Biology (CELL) Unit de Duve Institute Université catholique de Louvain Brussels 1200 Belgium; ^3^ Cellular and Molecular Pharmacology Unit (FACM) Louvain Drug Research Institute Université catholique de Louvain Brussels 1200 Belgium; ^4^ INM – Leibniz‐Institut für Neue Materialien gGmbH Campus D2 2 Saarbrücken 66123 Germany

**Keywords:** atomic force microscopy, cancer cells, cell mechanics, cholesterol, plasma membrane

## Abstract

Tumor cells present profound alterations in their composition, structural organization, and functional properties. A landmark of cancer cells is an overall altered mechanical phenotype, which so far are linked to changes in their cytoskeletal regulation and organization. Evidence exists that the plasma membrane (PM) of cancer cells also shows drastic changes in its composition and organization. However, biomechanical characterization of PM remains limited mainly due to the difficulties encountered to investigate it in a quantitative and label‐free manner. Here, the biomechanical properties of PM of a series of MCF10 cell lines, used as a model of breast cancer progression, are investigated. Notably, a strong correlation between the cell PM elasticity and oncogenesis is observed. The altered membrane composition under cancer progression, as emphasized by the PM‐associated cholesterol levels, leads to a stiffening of the PM that is uncoupled from the elastic cytoskeletal properties. Conversely, cholesterol depletion of metastatic cells leads to a softening of their PM, restoring biomechanical properties similar to benign cells. As novel therapies based on targeting membrane lipids in cancer cells represent a promising approach in the field of anticancer drug development, this method contributes to deciphering the functional link between PM lipid content and disease.

## Graphical abstract

Here, invasive malignant breast cancer cells are demonstrated to be softer than their healthy and premalignant counterparts and have an increased plasma membrane cholesterol content, increasing its tension and stiffness, which are critical parameters in the mediation of cellular responses.

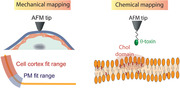

## 1 Introduction

Mammalian cells and in particular plasma membranes (PMs) have developed a vast palette of sense‐and‐respond pathways to react to physical stresses exerted by the environment in order to maintain cellular homeostasis. The cellular PM is a very dynamic system, which adjusts its biochemical and biophysical properties to maintain a stable equilibrium within a narrow range that is compatible with cellular physiology. Our understanding of the role of lipids in PMs radically changed over the last decades. While first thought to only possess a structural function, acting as a solvent that provides fluidity and elasticity to the membrane, more recent observations have dramatically expanded the role of lipids by showing that cells depend upon them for three main functions: energy storage, compartmentalization and signaling.^[^ ^1^ ^]^ Dysregulation of PM homeostasis as a result of changes in lipid composition and localization provides a favorable environment for the hyperactivation of various signaling networks (e.g., signaling by ErbB2), leading to oncogenesis.^[^ ^2^, ^3^, ^4^ ^]^ Oncogenesis is also often accompanied by changes in PM lipids composition, including higher levels of cholesterol in membrane, which led to intensive research of cholesterol metabolism in cancer during the last decade.^[^ ^5^ ^]^ It has been demonstrated that increased cholesterol biosynthesis and uptake can initiate or promote colon, breast, and prostate cancers.^[^ ^6^, ^7^ ^]^ Cholesterol is also known to covalently modify hedgehog family proteins^[^ ^8^ ^]^ and smoothened proteins,^[^ ^9^ ^]^ which modulate signaling pathways involved in tumorigenesis and cancer progression by facilitating the formation of specialized membrane microdomains, such as cholesterol‐enriched domains.^[^ ^10^ ^]^

Microscopy techniques are nowadays central to the elucidation of dynamics of domain formation and trafficking on complex cellular membranes. Cholesterol is typically detected with fluorescently labeled analogues or fluorescently labeled proteins having cholesterol binding domains, such as antibodies or toxin fragments.^[^ ^11^, ^12^, ^13^, ^14^, ^15^ ^]^ Although these techniques have so far provided valuable knowledge on the lipid membrane organization, further progress is currently hampered by their inherent limitations. First, these techniques require the use of bulky labels chemically foreign to the cell that can affect its functional state. Second, fluorescent probes can induce steric or electrostatic repulsion within the PM, which can lead to artifacts in the location and dynamics of cholesterol‐enriched domains. Third, excited fluorophores produce reactive oxygen species that oxidize proteins, nucleic acids, lipids and fluorophores, leading to photobleaching and cell cycle arrest or cell death. This highlights the importance of investigating membrane organization in physiologically relevant conditions. Tackling current limitations of microscopy techniques requires the development of integrated approaches, which can combine high‐resolution, vital imaging and physico‐chemical quantification of cell membranes.

**Figure 1:**
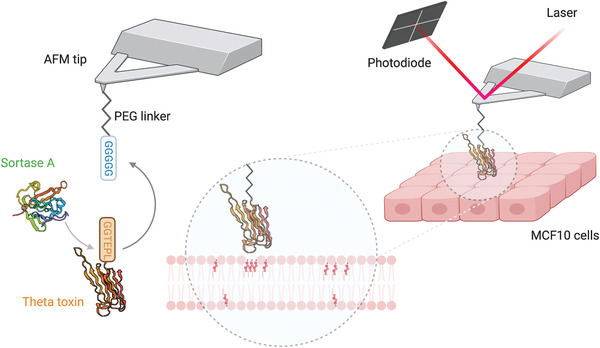
Probing *θ*‐toxin binding to living cells. Left) Schematic representation of the AFM tip functionalized with a PEG spacer fused to a hexaglycine peptide. The D4 fragment of the theta toxin is engineered to contain a LPETGG motif at its C‐terminal end. In a first step, the sortase A enzyme recognizes the LPETGG label and forms a thioester intermediate with the theta toxin fragment. The second step of the reaction is the nucleophilic attach by the hexaglycine motif and fusion between the theta toxin fragment and the AFM tip, with the regeneration of the sortase A enzyme. Right) The theta‐toxin decorated AFM tip scans the surface of MCF10 cells and binds cholesterol molecules exposed at the external leaflet of the plasma membrane. The position of the AFM tip and displacement of the cantilever are monitored by a laser beam reflected to a position‐sensitive photodiode.

Recent developments in atomic force microscopy (AFM) enabled it to simultaneously image and probe biochemical and mechanical properties of biological systems under physiological conditions and with no need for previous labeling.^[^ ^16^, ^17^ ^]^ In particular, force distance (FD) curve‐based AFM (FD‐AFM) approaches open new avenues to study the dynamic interplay between structure, function and nanomechanical properties of biological systems.^[^ ^18^, ^19^, ^20^, ^21^ ^]^ Using an AFM tip functionalized with the *C*‐terminal domain of *Perfringolysin O* toxin or theta (*θ*) toxin, a protein fragment that specifically targets cholesterol, we recently identified the organization and mechanical properties of cholesterol‐enriched lipid domains on lipid bilayers made of 1,2‐dioleoyl‐sn‐glycero‐3‐phosphocholine (DOPC) and cholesterol at the single‐molecule level.^[^ ^22^ ^]^ To this end, AFM tips were functionalized with polyethylene glycol (PEG) linkers terminated with hexaglycine motifs and covalently coupled to the *N*‐terminal part of LPETGG‐tagged *θ*‐toxin using sortase A (**Figure** **1**, left). Using this coupling strategy, we previously observed that cholesterol enriched domains within artificial membranes coincide with stiffer heterogeneities, enabling us to directly correlate chemical and mechanical properties at the single molecule level.^[^ ^22^ ^]^

Here, we used *θ*‐toxin AFM tips to study the organization and mechanical properties of cholesterol‐enriched domains directly on living cells for the first time (Figure 1, right). First, we extracted the mechanical properties of a series of MCF10 cell lines, as a model of breast cancer progression: MCF10A (benign), MCF10AT (premalignant, noninvasive), and MCF10CA1a (malignant, invasive). Our nanoscale analysis evidences a significant modification of the Young's modulus values with the degree of cell line malignancy. Using mechanical vertical segmentation, we observed a decrease of the Young's modulus of the cells (taking into account the influence of the cell cortex), while at the same time the stiffness of the PM increases. *θ*‐toxin derivatized AFM tips enabled us to evidence the PM cholesterol for the three cell lines. Excitingly, we observed that cholesterol content, at the PM external leaflet, increases with the degree of cell line malignancy, which translates into the observed stiffening of the PM. In addition, we noticed that cholesterol‐enriched assemblies locally contribute to cellular membrane stiffening. Our experiments provide direct quantitative evidence of how changes in PM cholesterol content contributes to modify cellular mechanical homeostasis. Our observations and methodology offer new perspective on the early detection of cancer risk factors and development of new anticancer therapies focused on cholesterol metabolism.

## 2 Results

### 2.1 Mechanical Phenotyping of Human Mammary Epithelial Cells

**Figure 2:**
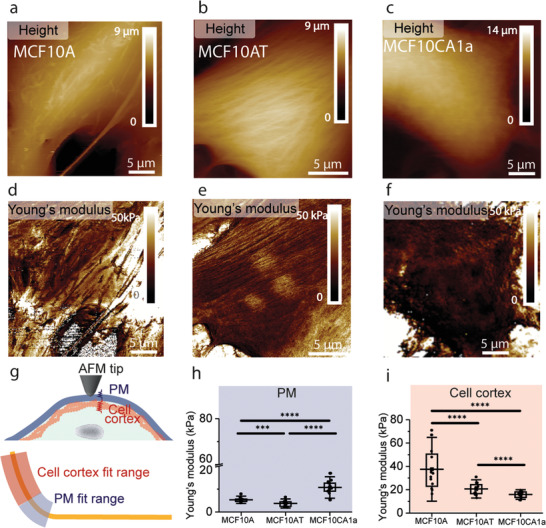
Mechanical phenotyping of human mammary epithelial cells. FD‐AFM topography images for a) MCF10A, b) MCF10AT, c) MCF10CA1a, and d–f) corresponding Young's modulus maps . g) PM and cell cortex elasticity of MCF10 cells. Schematic representation of an AFM tip indenting a MCF10 cell and contributions of PM (blue) and cell cortex (red) to the measured elastic properties. Hertz model was used to fit two different fit ranges of the repulsive part of FD curves (yellow line). PM contribution was defined for *δ* < 50 nm and cell cortex elasticity was extracted from *δ* between 50 and 200 nm. h) Malignant MCF10CA1a cells have stiffer PM than their healthy (MCF10A) and premalignant (MCF10AT) counterparts. i) Young's modulus of the cell cortex decreases with the progression of malignant properties. Each data point represents the mean Young's modulus value calculated for one cell. Box plots depict 25–75th percentiles, horizontal lines and centered squares show mean values and error bars indicate s.d. A number of 16 < *n* < 19 cells were analyzed from at least five independent experiments. Distributions in panel g) were evaluated using one‐way ANOVA followed by post‐hoc Tukey's HSD tests. ***p < 0.005 and ****p < 0.001.

The response of cancer cells to mechanical stress has been linked to their structural abnormalities, which led to their physical properties being pointed out as possible biomarkers for cancer cells classification.^[^ ^23^, ^24^, ^25^ ^]^ Mechanical properties of cancer cells have been used as biomarkers to detect cancer in early stages,^[^ ^26^, ^27^, ^28^, ^29^, ^30^ ^]^ so we tested this hypothesis with the MCF10 breast cell line series. We took advantage of AFM's capabilities of exploring cell mechanics with high spatial resolution^[^ ^31^, ^32^, ^33^ ^]^ and used multiparametric FD‐AFM to record simultaneous height and Young's modulus maps of MCF10 cells to compare their morphological and mechanical properties (Figure S1, Supporting Information). Height images in **Figure** **2**a–c shows that cell size varies between 30–40 µm and the maximum height at the central nuclear region is ≈9 µm for MCF10A and MCF10AT cells, while MCF10CA1a cells are higher (≈14 µm). A prominent feature of MCF10A and MCF10AT cells is the presence of aligned filamentous structures apparent in the Young's modulus map as long stiffer fibers (Figure 2d,e). On the other hand, MCF10CA1a cells display dispersed mesh‐like networks as main morphological feature and a loosely packed cytoskeletal network (Figure 2f). Our findings are in good agreement with previous studies where immunofluorescence assays of the cytoskeletal organization showed that cancer cell lines exhibit a weak localization of F‐actin structures in the cortical cytoskeleton, while healthy cells organize their actin in fibers.^[^ ^34^, ^35^, ^36^, ^37^ ^]^ In addition, recent research showed that cancer cells need to be softer and more deformable to invade surrounding tissue^[^ ^35^, ^38^ ^]^ and tumors with softer, larger cells at their periphery are more likely to spread.^[^ ^39^ ^]^ Malignant MCF10CA1a cells have increased invasion and migration capabilities^[^ ^40^, ^41^ ^]^ and we wondered whether mechanical properties of their cytoskeleton and the PM show any differences when compared to healthy MCF10A and premalignant MCF10AT cells. To tackle this point, we used an AFM‐based vertical segmentation approach on living cells, which enables us to isolate the contribution of the PM from overall cell mechanics (Figure 2g).^[^ ^32^, ^42^, ^43^ ^]^ We first extracted FD curves from the nuclear region of MCF10 cells probed with a bare AFM tip. Next, we defined different fit ranges in the contact region of these FD curves to specifically extract PM and cell cortex elasticity (Figure 2g and the Experimental Section). Outstandingly, we find the PM of MCF10CA1a cells (10.7 ± 3.1 kPa) shows a 2–3‐fold increase in Young's modulus as compared with MCF10A (5.3 ± 1.1 kPa) and MCF10AT (3.7 ± 1.3 kPa) (Figure 2h). We also analyzed the elastic properties associated to the cell cortex and found that mean Young's modulus values are in the range previously observed using multiparametric AFM imaging,^[^ ^32^, ^34^, ^44^ ^]^ as follows: 37.5 ± 18.2 kPa for MCF10A, 20.5 ± 5.0 kPa for MCF10AT, and 15.9 ± 2.7 kPa for MCF10CA1a, respectively (Figure 2i). We therefore observe a significant decrease of the cell cortex Young's modulus (≈57%) with the progression of malignant characteristics.

### 2.2 PM Cholesterol Detection Using *θ*‐Toxin Functionalized AFM Tips

**Figure 3:**
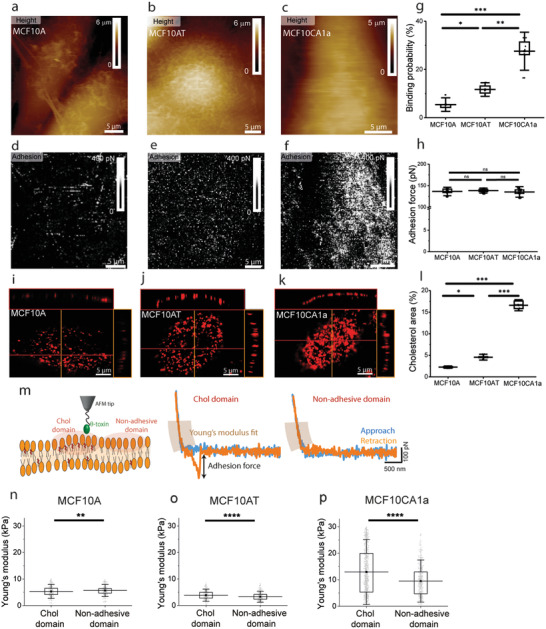
Plasma membrane cholesterol increases on malignant MCF10CA1a cells. a–c) FD‐AFM height images and d–f) corresponding adhesion maps for MCF10A (healthy), MCF10AT (premalignant), and MCF10CA1a (malignant) cells. g) Binding probability and h) adhesion forces for MCF10A, MCF10AT, MCF10CA1a cells, as extracted from adhesion maps. i–k) Confocal z‐stack images of cholesterol staining with mCherry‐*θ*‐toxin on the surface of MCF10 cells. The *x*–*y* cross sections and *x*–*z* reconstructions show that the focal plan chosen is on the cell surface exposed to AFM experiments. l) Cholesterol area graph for MCF10 cell lines extracted from CLSM images shows an increased presence of cholesterol as the malignant character of the cells progresses. m) A *θ*‐toxin derivatized AFM tip probes the surface of the cellular PM and encounters either cholesterol‐enriched domains where specific unbinding events are recorded (*F* > 100 pN), or areas lacking cholesterol where no adhesion events are observed (*F* < 30 pN). FD curves extracted from these regions were analyzed and Young's modulus values corresponding to the PM contribution (*δ* < 50 nm) were calculated. n–p) Elasticity of cholesterol and non‐adhesive domains for MCF10A, MCF10AT, and MCF10CA1a. A remarkable stiffening of cholesterol‐enriched domains with respect to non‐adhesive areas is observed on MCF10CA1a cells. Maps in panels (a–f) are representative for a number of 16 < *n* < 19 analyzed cells. Data points in panels (g,h,I) correspond to the mean values measured on a single cell (*n* ≥ 6 cells per condition). Data points in panels (n–p) correspond to Young's modulus values calculated from individual FD curves recorded on *n* = 5 cells per condition and at least 300 points are included in each graph. Box plots depict 25–75th percentiles, horizontal lines show mean values and error bars indicate s.d. Distributions in panels (g,h,I) were evaluated using one‐way ANOVA followed by post‐hoc Tukey's HSD tests. For panels (n,o,p) distributions were evaluated applying the Mann–Whitney U test. *p < 0.05, **p < 0.01, ***p < 0.005, ****p < 0.001 and n.s. non‐significant. All data is representative for at least five independent experiments.

We sought to determine whether the presence of cholesterol‐enriched domains and their distribution on the external leaflet of the PM can be evaluated in a label‐free manner with high spatial resolution on the surface of living cells. To this end, AFM probes derivatized with *θ*‐toxin were used to probe the presence of cholesterol‐enriched areas at the PM of living MCF10 cells (**Figure** **3**; Figure S2, Supporting Information).

To validate the specificity of the probed interactions, cells were exposed to methyl‐*β*‐cyclodextrin (M*β*CD), a pharmacological agent commonly used to remove membrane cholesterol by disrupting PM cholesterol‐enriched domains (Figure S2a–d, Supporting Information). Cholesterol depletion is broadly used to study the role of cholesterol in cellular processes and can be performed over days using inhibitors of its synthesis or acutely over minutes using chemical reagents. Acute cholesterol depletion by M*β*CD is the most widely used method to extract cholesterol from lipid membranes.^[^ ^45^ ^]^ Treatment with M*β*CD (2 × 10^−3^ m) for 2 h results in a drastic decrease of cholesterol content for MCF10AT and MCF10CA1a cells, but not MCF10A, as measured using an Amplex Red assay (Figure S3, Supporting Information). Shorter incubation times of 30 min and a higher M*β*CD concentration (10 × 10^−3^ m) rendered better results, with residual cholesterol percentages ranging between 50%–60% for the three cell lines (Figure S3, Supporting Information). Injection of M*β*CD (10 × 10^−3^ m) in the cell measurement medium significantly reduced the binding probability, as it can be observed in the adhesion maps displayed in Figure S2b,d, in the Supporting Information confirming the specificity of the interaction. Adhesive events were considered to be specific if they were detected at tip‐sample distances > 5 nm and when the adhesion force was > 80 pN (Figure S2e, Supporting Information). Additionally, each specific adhesion event was validated by fitting the extension profile of the PEG linker using the worm‐like chain model.^[^ ^46^ ^]^ Remarkably, our AFM‐based method enabled us to successfully probe for the first time cholesterol‐enriched areas on the surface of living cells with high spatial resolution, while working in physiological conditions and without the need of any labelling agents.

### 2.3 Plasma Membrane Cholesterol Content Increases on Malignant MCF10CA1a Cells

Multiparametric FD‐AFM height images and the corresponding adhesion maps reveal the location of specific adhesion events between the *θ*‐toxin bound to the AFM tip and cholesterol within the PM external leaflet (Figure 3a–f). Specific adhesion events between the tip and the cell surface are displayed as bright pixels on the adhesion maps in Figure 3d–f. We analyzed adhesion maps recorded over the surface of MCF10 cells and measured the binding probability as the percentage area covered by specific *θ*‐toxin‐cholesterol unbinding events (Figure 3g). The highest binding probability is observed on MCF10CA1a cells (27.5 ± 5.2%; *N* = 8), where cholesterol is densely distributed. On MCF10A and MCF10AT cells, we observe areas covered by adhesion events amounting 5.4 ± 1.8% (*N* = 7) and 11.6 ± 1.9% (*N* = 6) of the total, respectively.

The shape of cholesterol‐enriched areas is fairy irregular, which makes their size quantification a non‐trivial aspect of this analysis. To get more insights into the spatial organization of cholesterol assemblies on the surface of MCF10 cells, we analyzed the size of the adhesive domains in pixels where each pixel is ≈100 × 100 nm (Figure S4a, Supporting Information). Our analysis reveals that small domains of up to 10 pixels are the most abundant on the three cell types. However, we observed that the size of the domains increases with the degree of the malignancy. While MCF10A cells show domains extending up to 57 pixels, we observed domains up to 400 and 5000 pixels for MCF10AT and MCF10CA1a cells, respectively (Figure S4a, Supporting Information). We also quantified the nanomechanical properties of cholesterol‐enriched domains and extracted histograms showing the magnitude of the adhesion force, *F*, for each cell line (Figure 3h). We observe that the mean value of adhesion force remains reasonably constant on the three cell lines (*F*_MCF10A_ ≈ 141 ± 39 pN, *F*_MCF10AT_ ≈ 139 ± 64 pN, *F*_MCF10CA1a_ ≈ 129 ± 38 pN). This confirms the fact that we are measuring the same interaction between *θ*‐toxin and cholesterol on the surface of MCF10 cells.

To confirm that free cholesterol is present at the surface of MCF10 cells, we labelled cholesterol by incubating cells with mCherry‐*θ*‐toxin (1 × 10^−6^ m) during 30 min (Figure 3i–k) and images of the apical side of the cells were recorded in confocal laser scanning microscopy (CLSM) experiments. CLSM measurements reveal that the number of cholesterol assemblies increases with the metastasis level, confirming previous findings by AFM (Figure 3g). Sparsely distributed cholesterol assemblies of 300 nm in size can be observed on MCF10A cells. Premalignant MCF10AT cells display more frequent cholesterol assemblies, similar in size as the ones on healthy cells (Figure 3i–k). On the other hand, confocal images of malignant MCF10CA1a cells reveal a high abundancy of cholesterol assemblies with sizes ranging between 300 and 1200 nm. Cholesterol enrichment is observed on MCF10CA1a cells, covering almost 17% of the cell surface, while for MCF10AT and MCF10A cells the coverage is below 5% (Figure 3l). This is confirmed by the mean intensity of the fluorescence, which also displays higher values for MCF10CA1a, as compared to MCF10A and MCF10AT cells (Figure S4b, Supporting Information). Confocal microscopy experiments are in good agreement with AFM observations in terms of the spatial distribution and abundancy of cholesterol assemblies on the surface of MCF10 cells, however we observe that percentage of cholesterol area values (Figure 3l) are two times lower than those observed in AFM experiments (Figure 3g). On one hand, this could be a consequence of inherent instrumental limitations in CSLM, where the lateral resolution is dictated by the diffraction limit, reaching about 180 nm. In AFM, lateral resolution is primarily dictated by the radius of curvature at the end of the tip and on living cells it ranges between 50 and 100 nm. AFM can thus resolve smaller features on the cell surface and has higher sensitivity towards heterogeneities at the external leaflet of the PM. On the other hand, in CSLM experiments, *θ*‐toxin can localize cholesterol both at the internal and external PM leaflets, as well as in endocytic vesicles associated to the cell surface. Despite the difference in absolute values, both AFM and CSLM experiments confirm the presence of more cholesterol‐enriched assemblies at the surface of MCF10CA1a cells. AFM brings several advantages, such as increased resolution, lack of previous labeling and the ability to simultaneously extract mechanical information.

Given the increased presence of cholesterol‐enriched assemblies on the surface on MCF10CA1a cells (Figure 3g) and the higher Young's modulus of their PM (Figure 2g), we wondered whether cholesterol has a direct contribution to the mechanical resilience of the PM. While scanning the surface of MCF10 cells, the *θ*‐toxin derivatized AFM tip can encounter cholesterol‐enriched assemblies where specific unbinding events are recorded (*F* > 100 pN), or areas lacking cholesterol where FD curves display no adhesion events (*F* < 30 pN) are observed (Figure 3m). Young's modulus values corresponding to the PM contribution were calculated for these FD curves for the three MCF10 cell lines (Figure 3n–p). The elasticity of cholesterol and non‐adhesive domains is similar in the case of MCF10A cells (5.3 ± 1.7 kPa and 5.6 ± 1.5 kPa), while for MCF10AT cells a slight increase in Young's modulus is observed for cholesterol‐enriched domains (3.9 ± 1.5 kPa and 3.3 ± 1.3 kPa). A remarkable stiffening of cholesterol‐enriched domains with respect to non‐adhesive areas is observed on MCF10CA1a cells (13.1 ± 8.2 kPa and 9.0 ± 5.1 kPa).

### 2.4 Side‐to‐Side Comparison among Different Cell Lines

**Figure 4:**
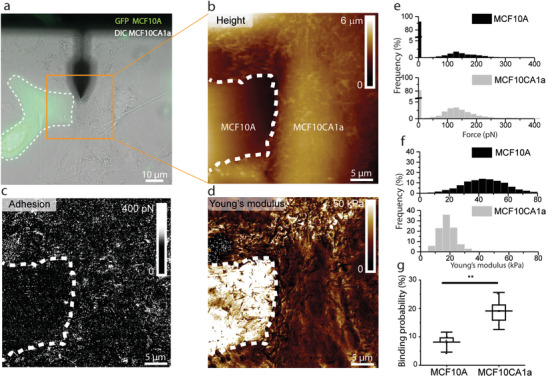
Comparison of cholesterol enrichment and elasticity of malignant and healthy cells co‐culture. a) Combined optical microscopy and FD‐AFM of adjacent MCF10A (healthy cell) and MCF10CA1a (malignant cell). a) Overlay of DIC and GFP signals of co‐cultured fluorescent MCF10A cells (cytoplasm GFP) and MCF10CA1a unlabeled. b) FD‐AFM height image and c) corresponding adhesion and d) Young's modulus maps obtained by probing adjacent cells indicated in the orange square in a with a *θ*‐toxin AFM tip. The adhesion map shows higher interactions on MCF10CA1a (malignant cell, white pixels) and the Young's modulus map shows higher values for MCF10A (healthy cell). Histograms of e) adhesion forces and f) Young's modulus distributions extracted from the corresponding maps of MCF10A and MCF10CA1a cells. g) Binding probabilities extracted from co‐cultured MCF10A and MCF10CA1a cells. Higher binding probabilities are observed for MCF10CA1a cells, as compared to MCF10A. Data points in panel g correspond to the mean values measured on a single cell (*n* = 5 cells per condition). Box plots depict 25–75th percentiles, horizontal lines show mean values and error bars indicate s.d. Distributions in panel g were evaluated using one‐way ANOVA followed by post‐hoc Tukey's HSD tests. **p < 0.01. All data is representative for a number of five independent experiments.

As we evidenced that MCF10CA1a cells are more compliant and show alteration in their cholesterol content and organization, we wanted to further evidence this difference by a side‐to‐side comparison, enabling a direct internal control experiment. To this end, we co‐cultured MCF10A and MCFC10A1a cell lines. Combined optical and multiparametric FD‐AFM experiments were conducted using living fluorescently labeled MCF10A cells (green) and unlabeled MCF10CA1a cells (**Figure** **4**). Using AFM tips functionalized with *θ*‐toxin, a confluent monolayer of co‐cultured MCF10A and MCF10CA1a cells was imaged using conditions to propagate both cell types (Figure 4a–d). Driven by the intensity of the fluorescence, we chose fields of view in which both types of cells were adjacent. Adhesion maps (Figure 4c) recorded with a *θ*‐toxin functionalized AFM tip showed a higher density of adhesion events on MCF10CA1a, as confirmed by the extracted histogram in Figure 4e. The adhesion forces peak at similar values of 142 ± 34 pN for MCF10A and 133 ± 30 pN for MCF10CA1a, which confirms the hypothesis that we are measuring the same interaction. The Young's modulus map (Figure 4d) and the extracted histograms (Figure 4f) evidence higher values of Young's modulus for MCF10A cells (43.9 ± 12.4 kPa) in comparison to MCF10CA1a cells (10.6 ± 4.6 kPa). Again, this direct side‐to‐side observation reinforces our previous data obtained on the isolated cultures, which pointed out the malignant cells as softer than the healthy and premalignant ones. We analyzed adhesion maps of co‐cultured MCF10A and MCF10CA1a cells and compared the binding probabilities in Figure 4g. The twofold difference in binding probability between MCF10CA1a and MCF10A supports our previous findings regarding the increased presence of cholesterol‐enriched areas on the surface of malignant cells. These experiments prove that our approach for the detection of cholesterol‐enriched assemblies on the surface of living cells is not biased by the local topographical or mechanical heterogeneities of the cell surface.

### 2.5 Cholesterol Detection Is Independent from Cytoskeletal State

**Figure 5:**
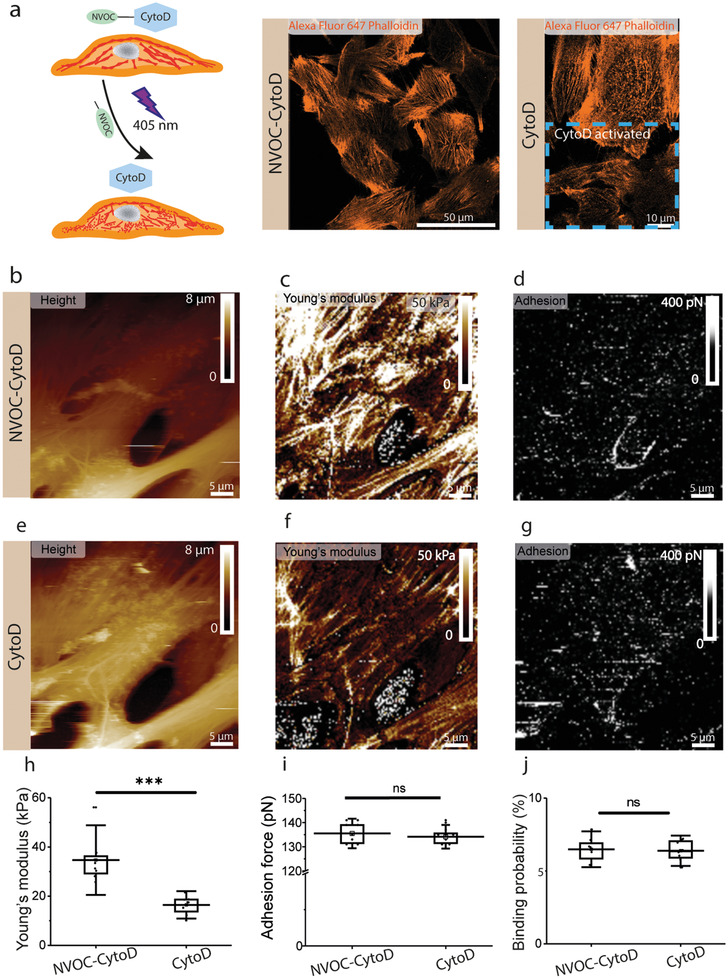
Cytoskeletal mechanics does not have impact on cholesterol detection. a) Left: schematic representation of Cytochalasin D photoactivable operating mode. Cythochalasin D is linked to a protecting NVOC group, which makes it inactive. When NOVOC‐Cytochalasin D is illuminated with a UV laser at 405 nm wavelength, the bonds between NVOC and Cytochalasin D are cleaved and the drug becomes activated. Activated Cytochalasin D is able to depolymerize actin filaments, which makes cells softer. Right: confocal images of actin filaments staining for MCF10A cells. Blue dashed rectangle corresponds to the area illuminated by the UV laser. Confocal images show a strong decrease of actin filaments inside this area where Cytochalasin D was activated. b,e) FD‐AFM height images and corresponding c,f) Young's modulus and d,g) adhesion maps for MCF10A before (b,c,d) and after actin depolymerization (e,f,g). h) Young's modulus, i) adhesion force and j) binding probability for MCF10A cells before and after Cytochalasin D treatment. The Young's modulus data shows cells display lower rigidity after Cytochalasin D treatment, while the magnitude and frequency of the adhesive events do not change significantly. Data points in panels h‐j correspond to the mean values measured on a single cell (*n* = 8 cells). Box plots depict 25–75th percentiles, horizontal lines show mean values and error bars indicate s.d. Distributions in panels h,i,j were evaluated using one‐way ANOVA followed by post‐hoc Tukey's HSD tests. ***p < 0.005 and n.s. non‐significant. All data is representative for a number of eight independent experiments.

As we observed a higher binding probability on the softer MCF10CA1a cells, we wanted to test if this observation could originate from the lower cell cortex Young's modulus, which translates into a higher tip‐sample contact area for the same applied force and could potentially enhance the binding probability. To test this hypothesis, we conducted independent control experiments using Cytochalasin D, a cell‐permeable and potent inhibitor of actin polymerization that disrupts actin microfilaments (**Figure** **5**a). For this experiment, we used nitroveratryloxycarbonyl (NVOC)‐Cytochalasin D, a photoactivated drug able to depolymerize the actin filaments in the cytoskeleton.^[^ ^47^, ^48^ ^]^ Actin depolymerizing drugs were previously shown to have an effect on the elasticity of living cells.^[^ ^49^ ^]^ To check the efficiency of NVOC‐Cytochalasin D in depolymerizing the actin cytoskeleton, we incubated the drug with MCF10A cells and activated it by illuminating a defined area with the UV laser, as shown in Figure 5a. Next, cells were fixed and actin filaments were stained with Alexa Fluor 647 phalloidin. We recorded confocal images and we observed the actin fibers are not visible in the illuminated area (Figure 5a), which confirms the efficiency of the drug. In a different set of experiments, we recorded AFM multiparametric height, Young's modulus and adhesion maps before and after Cytochalasin D activation on live MCF10A cells (Figure 5b–g). We observed that while the cell morphology does not change, the cell Young's modulus is reduced by ≈50% from 34.7±9.4 kPa before to 16.4±3.7 kPa after Cytochalasin D activation (Figure 5c,f,h). Filamentous structures are still visible after actin depolymerization, though to a lower extent. Remarkably, binding properties are not altered by the Cytochalasin D activation, as we observed similar binding probabilities (6.5 ± 1.2% before and 6.4 ± 0.7% after treatment) and similar adhesion forces (from 135 ± 4 pN before to 134 ± 3 pN after treatment) (Figure 5d,g,i). Notably, Cytochalasin D‐treated MCF10A cells have similar mechanical properties as malignant MCF10CA1a cells (≈16 kPa), but the PM cholesterol content does not change. Altogether, these experiments confirm that our method is able to detect cholesterol‐enriched areas at the surface of the PM and that the molecular recognition process involved is decoupled from cellular cortex mechanical properties.

### 2.6 Cholesterol Depletion Reduces Binding Probability and Changes PM Mechanics

**Figure 6:**
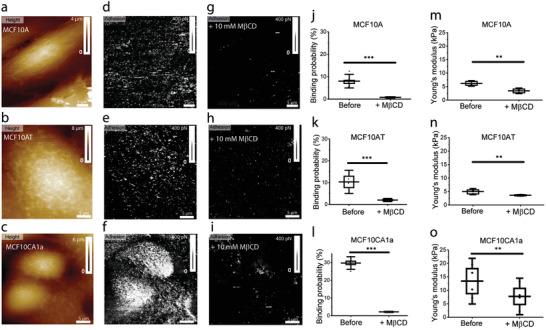
Cholesterol depletion affects plasma membrane mechanics. a–c) FD‐AFM height images and corresponding adhesion maps before d–f) and after g–i) cholesterol depletion with 10 × 10^−3^ m M*β*CD for MCF10A, MCF10AT, and MCF10CA1a. j–l) Binding probability box plots for MCF10A, MCF10AT, and MCF10CA1a confirm a decrease in *θ*‐toxin‐cholesterol adhesive events after adding 10 × 10^−3^ m M*β*CD. m–o) Young's modulus of elasticity extracted from maps recoded on MCF10A, MCF10AT, and MCF10CA1a cells following cholesterol depletion with 10 × 10^−3^ m M*β*CD. FD curves were analyzed and Young's modulus values corresponding to the PM contribution (*δ* < 50 nm) were calculated. A softening of the PM is observed for all three cell lines as a result of M*β*CD treatment. Data points in panels j‐o correspond to the mean values measured on a single cell during *n* ≥ 3 independent experiments. Box plots depict 25–75th percentiles, horizontal lines show mean values and error bars indicate s.d. Distributions in panels j‐o were evaluated using one‐way ANOVA followed by post‐hoc Tukey's HSD tests. **p < 0.01 and ***p < 0.005.

Finally, we wanted to determine whether surface cholesterol content could influence cell mechanics. To test this, we analysed both the cholesterol content at the cell surface by AFM and the PM Young's modulus before and after treatment by M*β*CD (10 × 10^−3^ m) for 30 min, used to deplete cholesterol (**Figure** **6**a–i). We noticed a strong decrease of the binding probability after M*β*CD treatment (Figure 6d–i): from 8% to 0.7% for MCF10A, from 10.3% to 2% for MCF10AT and from 29.7% to 2.2% for MCF10CA1a (Figure 6j–l).

Simultaneously to the cholesterol content, we also analyzed by AFM the Young's modulus values of the PM of MCF10A cells before and after treatment with M*β*CD (Figure 6m–o). A significant effect is observed on the three types of cells. Young's modulus values show a remarkable decrease of 44% for MCF10A cells (from 6.1 ± 0.7 kPa to 3.4 ± 0.7 kPa) and 42% for MCF10CA1a cells (from 13.4 ± 5.6 kPa to 7.7 ± 4.5 kPa). Cholesterol depletion has a less marked effect on MCF10AT cells, which show a 26% decrease (from 4.9 ± 0.7 kPa to 3.6 ± 0.2 kPa). The effect of cholesterol depletion on lipid packing and cell stiffness was previously observed on aortic endothelial cells, cardiomyocytes, HeLa, and red blood cells.^[^ ^50^ ^]^ These observations suggest that cholesterol depletion leads to a decrease in membrane tension, modifications in plasma membrane‐cytoskeleton connections and underlying cytoskeleton response to mechanical stress.^[^ ^50^ ^]^

## 3 Discussion

Breast cancer occurs with the highest incidence among women worldwide and is curable in ≈70–80% of patients with early‐stage, non‐malignant disease. On the molecular level, breast cancer is a heterogeneous disease and generally divided into various types according to the expression status of estrogen receptors, progesterone, and ErbB2.^[^ ^51^ ^]^ Cancer progression is generally associated with alterations in cellular responses to both chemical and mechanical signals. Changes in membrane lipid composition represents a landmark of numerous cancers. For instance, phospholipid and fatty acid profiles are altered in breast cancer, which is linked to altered cell proliferation that requires the activation of the fatty acid biosynthesis in order to provide enough building blocks to from new membranes. Cholesterol, as the major sterol component of cell membrane, accounts for about 10–40 mol% of the PM lipids and plays a pivotal role maintaining the structural integrity and regulating the fluidity of cell membrane. When cholesterol levels are maintained below 15%, PM lipids are found in different degrees of a liquid‐disordered phase. An enrichment in cholesterol above 20% leads to an increase in membrane packing in a more rigid liquid‐ordered phase.^[^ ^52^ ^]^ This is due to the rigid ring structure of cholesterol that considerably reduces the *cis*‐*trans* isomerization of adjacent unsaturated lipid acyl chains and therefore orders them, resulting in a reduction of their dynamics and fluidity. The increased viscosity of cholesterol‐containing membranes slows down the lateral diffusion and fast rotational movements of lipids and embedded membrane proteins.^[^ ^53^, ^54^ ^]^ Cholesterol content therefore also directly contributes to the homeodynamics of various membrane proteins on the cell surface. Basic research has shown that increased cholesterol is a metabolic signature in breast cancer and its accumulation is positively correlated with advanced clinical staging and metastasis.^[^ ^5^, ^55^, ^56^ ^]^ Cholesterol promotes breast cancer via several mechanisms, involving an interplay among modified lipoproteins, proinflammatory signaling pathways, and breast cancer tumorigenic processes. However so far, the connection between cholesterol content and the physical properties of breast cancer cells are poorly understood mainly due to the lack of appropriate techniques enabling to study the PM‐associated cholesterol content and mechanics of breast cancer cell directly on living cells. A better understanding at the mechanistic level of the role of the PM in cellular mechanotransduction and metastasis development will push the mechanotyping of cells for diagnosis and treatment purposes a step further.

In this study, we investigated a series of MCF10 cell lines, as a model of breast cancer progression, to determine how membrane cholesterol distribution is altered during breast cancer development and to understand the connection between cholesterol levels and physical properties of breast cancer cells and their PM.

In particular, we compared MCF10A (benign), MCF10AT (premalignant, noninvasive), and MCF10CA1a (malignant, invasive) cells. FD‐AFM with *θ*‐toxin derivatized tips was used for the first time to highlight more recently appreciated roles for cholesterol in cancer cells development, by studying these cell lines in physiologically relevant conditions without any fixation or labelling steps. In a first step, we used an AFM‐based vertical segmentation approach on living cells to extract their mechanical properties. Our results evidence a significant decrease of the overall Young's modulus values with the degree of cell line malignancy. Remarkably, this overall softening is accompanied by a stiffening of the PM, which we hypothesized to result from an increase of PM‐bound cholesterol abundance (Figure 2). To put this in evidence, we used AFM tips functionalized with *θ*‐toxin and imaged the PM‐associated cholesterol. We successfully probed cholesterol‐enriched lipid assemblies on the PM surface of the three cell lines and found that cholesterol content detected at the external leaflet of the PM increases with the degree of the cell line malignancy.

## 4 Conclusions

**Figure 7:**
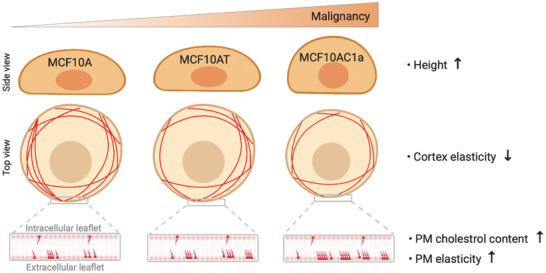
Cholesterol‐enrichment at the PM surface correlates with a stiffening of the cell membrane and oncogenesis. A series of breast cancer cell lines (healthy MCF10A, pre‐malignant MCF10AT, and malignant MCF10CA1a) was used to study the relationship between dysregulation of cholesterol content, mechanical resilience and malignancy level. Malignant MCF10CA1a cells are less spread on the surface and a significant decrease of their cellular Young's modulus is observed when compared with their healthy and premalignant counterparts. Cholesterol content at the external leaflet of the PM increases with the degree of cell line malignancy and contributes to the stiffening of the PM.

Physical forces play a key role in cancer progression and treatment. We demonstrated that malignant MCF10CA1a cells are softer and the properties of their cellular PM are profoundly altered, showing an increased amount of cholesterol that enhances membrane tension and stiffness, by filling the gaps between lipid acyl chains (**Figure** **7**). We showed how the link between cell functional state and mechanical response can provide powerful insights into the mechanism by which cholesterol enrichment confers drug resistance of malignant cells and enhances tumor progression. To best of our knowledge, this is the first time that the contribution of cholesterol to plasma membrane mechanical resilience is quantified in situ in a label‐free setup, on living cells and in a non‐invasive manner. Considering the key role of cholesterol in breast cancer development, our technique offers new perspectives to directly test molecules targeting cholesterol enrichment and membrane modulation on living cells. Lipid therapy is becoming a very interesting alternative and combining it with available cancer treatments brings new opportunities for preventing the deleterious effects of high cholesterol in breast cancer.

## 5 Experimental Section

##### Culture of Cell Lines

MCF10A and MCF10AT kindly offered by Prof. Pierre Sonveaux (UCLouvain, Brussels, Belgium) were grown in DMEM/F12 (Thermofisher) supplemented to contain horse serum (5%, Thermofisher), CaCl_2_ (1.1 × 10 ^−9^ m), Insulin (10µg mL^−1^, Sigma,), Human EGF (2ng mL^−1^, Peprotech: AF‐100‐15), Hydrocortisone (0.5µg mL^−1^, Sigma), penicillin (100 U mL^−1^), and streptomycin (100 µg mL^−1^, Invitrogen) at 37 °C in a humidified atmosphere with CO_2_ (5%). MCF10CA1a were grown in DMEM/F12 (Thermofisher) supplemented to contain (5%, Thermofisher), CaCl_2_ (1.1 × 10 ^−9^ m), penicillin (100 U mL^−1^) and streptomycin (100 µg mL^−1^) at 37 °C in a humidified atmosphere with CO_2_ (5%). Cells were further used for AFM and CSLM experiments when reaching 80% confluence levels.

##### Live Cell Staining

Live cells (MCF10A) were fluorescently labeled with CellTracker (Invitrogen) to label cytoplasm. The cells were imaged by using excitation of a 488 nm laser on CLS microscope (Zeiss). For actin filaments staining, cells were fixed with a solution of paraformaldehyde (4%, Merck) for 15 min and washed three times in PBS. Cells were then incubated for 45 min at 37 °C with Alexa Fluor 647 phalloidin (Invitrogen, 1:200). For cholesterol staining, cells were incubated 30 min with purified Theta‐toxin (1 × 10^−6^ m) in BSA (1 mg mL^−1^) at 20 °C and washed 2 times with DMEM/F12.^[^ ^57^ ^]^

##### Functionalization of AFM Tips

NHS‐PEG27‐acetal linkers were used to functionalize AFM probes.^[^ ^58^ ^]^ AFM tips (PFQNM‐LC, Bruker) were first immersed in chloroform for 10 min dried with a stream of filtered nitrogen, cleaned for 10 min using an ultraviolet radiation and ozone (UV‐O) cleaner (Jetlight) and incubated during 2 h in a desiccator under argon with a tray with APTES and another tray with triethylamine (30 µL APTES and 10 µL triethylamine). After removing the APTES and trimethylamine trays, the tips were left inside the dessicator for 2 days to cure the APTES coating. To ensure a low grafting density of the linker on the AFM tip, acetal‐PEG24‐NHS (3.3 mg) was diluted in chloroform (0.5 mL) and trimethylamine (30 µL). The cantilevers were immersed for 2 h in this solution, washed three times with chloroform, and dried with nitrogen. Cantilevers were then immersed for 1 h in Gly_10_Lys (1 × 10^−3^ m) and washed three times with milliQ water. The tips were then immersed for 1 h at 37 °C in a freshly prepared solution of *θ*‐toxin (10 × 10^−6^ m) and sortase A (10 × 10^−6^ m), washed three times with Tris‐buffer (50 × 10^−3^ m Tris, 150 × 10^−3^ m NaCl, 10 × 10^−3^ m CaCl_2_, pH 7.5) and stored at 4 °C in the same buffer. Cantilevers were used in AFM experiments the same day they were functionalized.

##### AFM Imaging and Fluorescence Microscopy On Living Cells

AFM images of confluent layers of MCF10 cells were acquired using an AFM (Bioscope Resolve, Bruker) operated in the PeakForce QNM mode (Nanoscope software v9.2) and coupled to an inverted epifluorescence microscope (Zeiss Observer Z.1) or a confocal laser scanning microscope (Zeiss LSM 900). A 40× oil objective (NA = 0.95) was used. The AFM was equipped with a 150 µm piezoelectric scanner and a cell‐culture chamber allowing to control the temperature, the humidity and the CO_2_ concentration.^[^ ^59^ ^]^ Overview images of cell surfaces (20–50 µm^2^) were recorded at imaging forces of 500–750 pN using PFQNM‐LC probes (Bruker) having tip lengths of 17 µm, tip radii of 65 nm and opening angles of 15°. All fluorescence microscopy and AFM imaging experiments were conducted under cell‐culture conditions using the combined AFM and fluorescence microscopy chamber at 37 °C in DMEM/F12 medium. A gas mixture of synthetic air with CO_2_ (5%) at 95% relative humidity using a gas humidifier membrane (PermSelect silicone) was infused at 0.1 L min^−1^. into the microscopy chamber. The humidity was controlled using a humidity sensor (Sensirion). Cantilevers were calibrated using the thermal noise method,^[^ ^60^ ^]^ yielding values ranging from 0.08 to 0.14 N m^−1^ for PFQNM‐LC probes. The AFM tip was oscillated in a sinusoidal fashion at 0.25 kHz with a 750 nm amplitude in the PeakForce Tapping mode. The sample was scanned using a frequency of 0.125 Hz and 128 or 256 pixels per line (256 lines).

##### Confocal Microscopy Imaging

MCF10A cell lines (MCF10A, MCF10AT, MCF10CA1a) were cultured or co‐cultured on a 47‐mm glass‐bottomed petri dish (WillCo Wells) for 1 or 2 days before the experiment to ensure formation of a confluent monolayer on the day of the experiment. Cells were imaged by confocal laser scanning microscopy using a Zeiss LSM 900 microscope with a 647 nm laser for Alexa Fluor 647 and a 488 nm laser for CellTracker green and a ×40 oil objective (NA = 0.95). All experiments were conducted at room temperature with cells maintained in DMEM/F12 culture medium and a gas mixture of synthetic air with 5% CO_2_ at 95% relative humidity that was infused at 0.1 L min^−1^. into the microscopy chamber using a gas humidifier membrane (PermSelect silicone). The humidity was controlled using a humidity sensor (Sensirion). During recording, the focus was kept constant on the upper surface of cells. *θ*‐toxin confocal images were recorded with a Zeiss LSM 510 confocal microscope equipped with a 63× water objective and FluoRed laser AF555. Fluorescence images were exported as 12‐bit TIFF files and further processed using ImageJ (National Institutes of Health, Bethesda).

##### Actin Filaments Depolymerization

NVOC‐Cytochalasin D was added to the MCF10A cells medium at a final concentration of 50 × 10^−6^ m. A chosen area was illuminated by a UV 405 nm laser (actual laser power of 0.02 mW) during 3 pulses of 45 s to dispense the drug. AFM images were recorded after 15 min. To confirm that actin filaments were depolymerized, at the end of the AFM experiment, cells were fixed with a solution of paraformaldehyde (4%, Merck) for 15 min and washed three times in PBS. Fixed cells were further incubated with Alexa Fluor 647 phalloidin (Invitrogen, 1:200) and imaged with CLSM.

##### Cholesterol Depletion and Measurement

MCF10A, MCF10AT, and MCF10CA1a were treated with M*β*CD (10 × 10^−3^ m, Sigma‐Aldrich) in DMEM/F12 medium without serum with Bovine Serum Albumin (1 mg mL^−1^, fatty acid free) (Sigma‐Aldrich) at 37 °C. After 30 min, AFM images were recorded or cells were lysed in order to quantify residual cholesterol. To this aim, trypsin was added to the cells until they were detached. DMEM/F12 was supplemented to inactivate the trypsin. To lyse the cells, the cell suspension was diluted (1 × 10^6^ cells mL^−1^) in NaCl (0.9%) and submitted to 2 cycles of freezing/thawing at −80 °C and vortexed. To extract residual cholesterol, chloroform/methanol (3 mL, 2:1 volume ratio) was added to the cell lysate (0.8 mL). The solution was vortexed and centrifuged for 15 min at 3000 rpm at room temperature. The organic phase located at the bottom was aspirated and washed with NaCl (0.05 m). Then, the washed organic phase was vortexed and centrifuged 15 min at 3000 rpm. The aqueous phase located on the surface were removed. Then, the solution was washed again with CaCl_2_ (0.36 m/methanol, 1:1), vortexed and centrifuged 15 min at 3000 rpm. The aqueous phase was also removed and a new wash with CaCl_2_ (0.36 m/methanol, 1:1) was performed. After, Triton X‐100 (1%) was added to the organic phase. The sample solution was evaporated and resuspended in H_2_O. Finally, cholesterol was assessed with Amplex Red cholesterol assay kit.

##### Data Analysis

AFM images were analyzed using the Nanoscope Analysis software (v1.9, Bruker) and ImageJ (v1.52e). Optical images were analyzed using Zen Blue software (Zeiss). Raw FD curves extracted from multiparametric FD‐AFM maps were processed offline using the AtomicJ open source software.^[^ ^61^ ^]^

To correct for the tilt sometimes present in raw FD‐curves, the baseline of the retraction curve was corrected using a 2nd degree polynomial fit on the off‐contact area. To extract Young's modulus values, Hertz's model for a sphere was fit to the contact region of retraction part of FD‐curves^[^ ^62^, ^63^ ^]^

(1) $$\begin{array}{c} F^{2/3}={\left(\frac{4}{3}\;\frac{E}{\left(1-\nu^{2}\right)}\;\sqrt{R}\right)}^{2/3}\;\delta \; \end{array}$$

where *E* is the Young's modulus, *δ* is the indentation depth, *ν* is the Poisson ratio, and *R* is the contact radius. A Poisson's ratio value of 0.5 was used. Young's modulus was computed from the slope of Equation (1). Two indentation depth ranges were defined: ∆*δ* < 50 nm corresponding to PM contribution and ∆*δ* > 50 nm corresponding to the cell cortex. A correction for the effect of the substrate was implemented for the estimation of the PM Young's modulus using a spherical indenter^[^ ^64^ ^]^

(2) $$\begin{array}{c} F\;=\;\frac{16E}{9}\sqrt{R}\delta^{3/2}\left[1+0.884\alpha +0.781\alpha^{2}+0.386\alpha^{3}+0.0048\alpha^{4}\right] \end{array}$$

where $\alpha \;=\;\frac{\sqrt{R\delta }}{h}$ and *h* is the thickness of the layer.

The retraction part of FD‐curves was also analyzed to measure specific unbinding events between cholesterol‐enriched assemblies and the *θ*‐toxin derivatized AFM tip. An event was counted as specific when the minimum adhesion force was higher than 80 pN and the unbinding distance was more than 5 nm away from the contact point. The noise level in raw FD‐curves was calculated by calculation the standard deviation from a linear fit of the off‐contact part of the retraction curve. Typical noise level values were below 20 pN.

##### Statistical Analysis

Statistical tests were carried out in Prism (GraphPad) and Origin (OriginLab). Differences between means of data with normal distributions or a small sample set were determined by using an analysis of variance (ANOVA) followed by Tukey's post‐hoc test. Data displaying non‐normal distributions was analyzed using a two‐tailed Mann‐Whitney U test. In all statistical analysis, *p* values are given as not significant (ns) if *p* >0.05 and gradually indicated as significant if *p* < 0.05 (*), *p* < 0.01 (**), *p* < 0.005 (***), and *p* < 0.001 (****).

## Conflict of Interest

The authors declare no conflict of interest.

## Supporting information

Supporting Information
