# Supplementary material for: Label‐Free Imaging of Cholesterol Assemblies Reveals Hidden Nanomechanics of Breast Cancer Cells
Source: Adv Sci (Weinh). 2020 Oct 8;7(22):2002643. doi: 10.1002/advs.202002643 (PMC7675049; doi:10.1002/advs.202002643)
Supplement: Supplementary file 1 — Supporting Information [file ADVS-7-2002643-s001.pdf]

© 2020 Wiley-VCH GmbH

ADVANCED SCIENCE
Open Access

Supporting Information

for *Adv. Sci.,* DOI: 10.1002/advs.202002643

Label-Free Imaging of Cholesterol Assemblies Reveals Hidden Nanomechanics of Breast Cancer Cells

*Andra C. Dumitru, Danahe Mohammed, Mauriane Maja, Jinsung Yang, Sandrine Verstraeten, Aranzazu del Campo, Marie-Paule Mingeot-Leclercq, Donatienne Tyteca, and David Alsteens**

Supporting Information

Label-free imaging of cholesterol assemblies reveals hidden nanomechanics of breast cancer cells

*Andra. C. Dumitru[#], Danahe Mohammed[#], Mauriane Maja, Jinsung Yang, Sandrine Verstraeten, Aranzazu del Campo, Marie-Paule Mingeot-Leclercq, Donatienne Tyteca, David Alsteens**

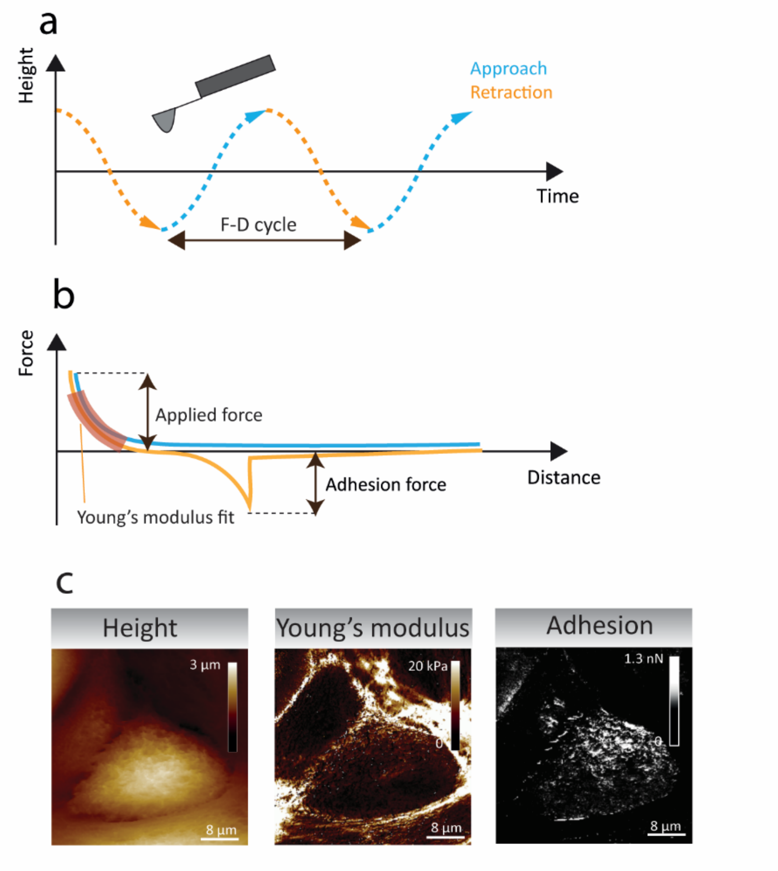

Figure S1. Principle of using FD-AFM to image and map biophysical properties of living cells. (**a**) The operating principle of FD-AFM is based on a cantilever being oscillated well below its resonance frequency, while using the tip-sample interaction force (peak force) as feedback. (**b**) The tip is continuously approached and retracted from the sample and from each FD cycle the sample height is determined and tip-sample interactions are analyzed. The adhesion force is calculated from the retraction part of the FD curve, as the minimum force acting on the cantilever before going back to its initial position. Quantitative parameters, such as Young's modulus, can be extracted by fitting the part of the FD curve where the tip is in contact with the sample (red shaded area) with contact mechanics models for elasticity. (**c**) Biophysical properties of living cells (Young's modulus and adhesion) are extracted, mapped pixel-by-pixel and directly correlated to the topography (height map).

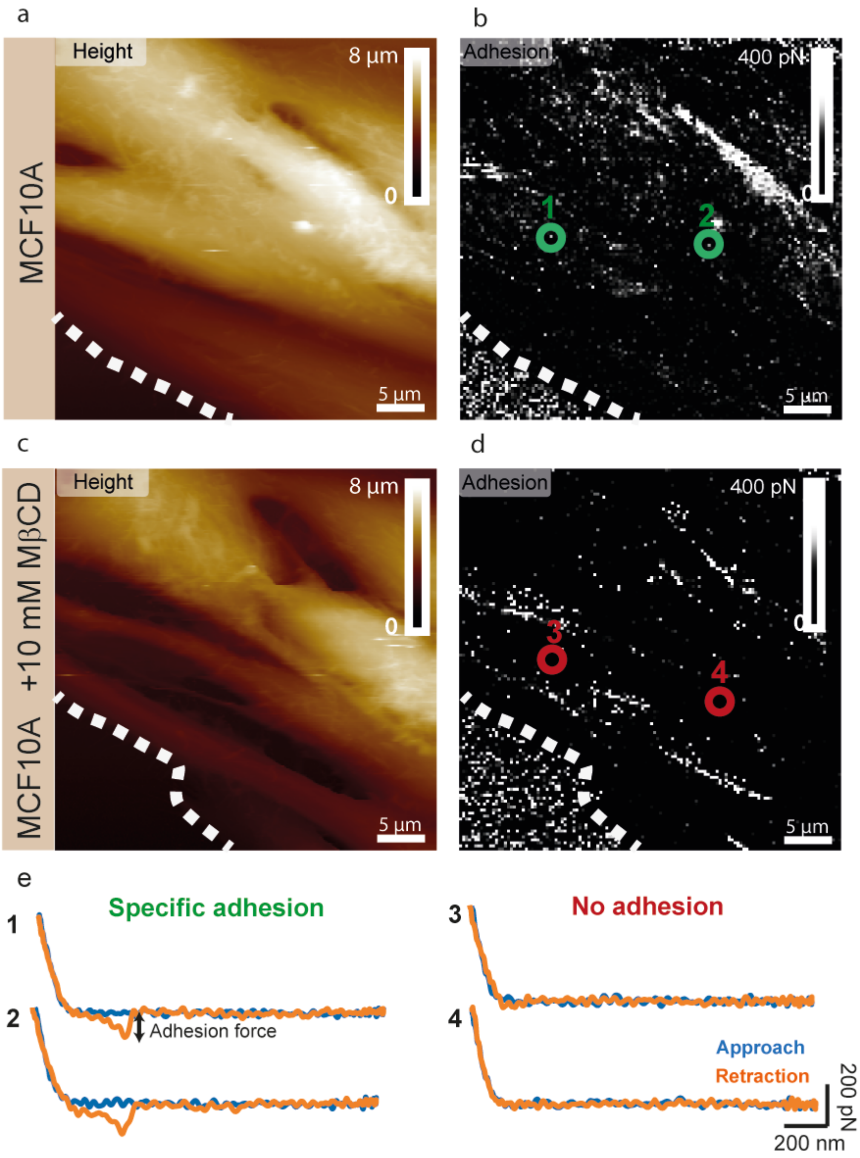

Figure S2. Cholesterol depletion confirms the specificity of the interaction between θ-toxin and cholesterol. (**a**) AFM topography image of MCF10A cells immobilized on a glass Petri dish and (**b**) adhesion map recorded simultaneously showing that the θ-toxin functionalized tip interacts with the cellular PM. (**c**) AFM topography image of the same area in (a) recorded after the injection of 10 mM MβCD in the imaging buffer. (**d**) Adhesion map corresponding to the topography in (**c**) showing a drop in binding frequency as a result of cholesterol depletion by 10 mM MβCD. (**e**) Representative FD curves showing either specific adhesion events (curves 1-2) or no adhesion (curves 3-4) were extracted from the encircled areas marked in the adhesion maps (**b**) and (**d**). Dotted lines represent the border between the cell and the glass substrate.

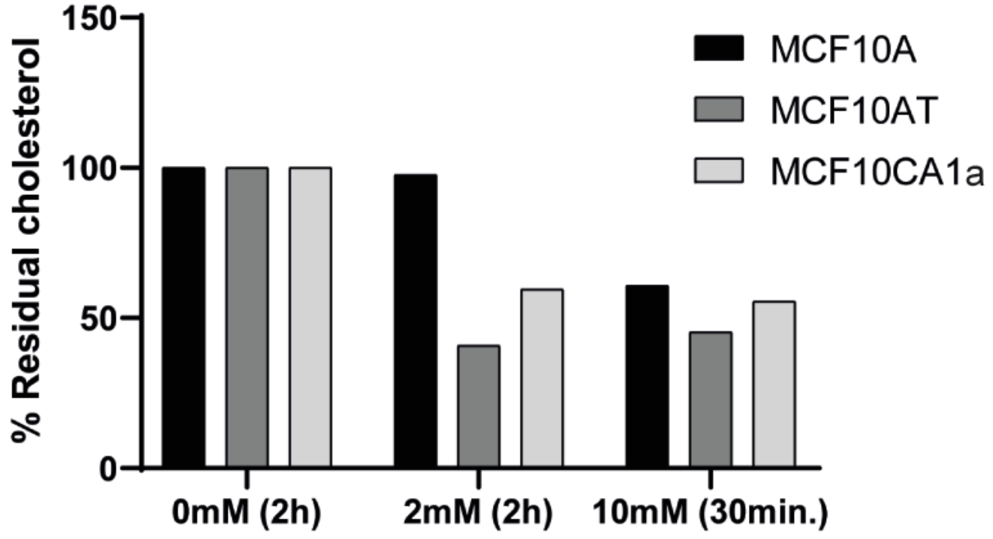

Figure S3. Residual Cholesterol after MβCD depletion. MCF10A (black), MCF10AT (dark gray) and MCF10CA1 (light gray) were treated with MβCD 2mM during 2h and 10mM during 30 min and the percentage of residual cholesterol was measured after extraction using an Amplex Red assay. Data is expressed as % of control and is the cholesterol: protein content ratio.

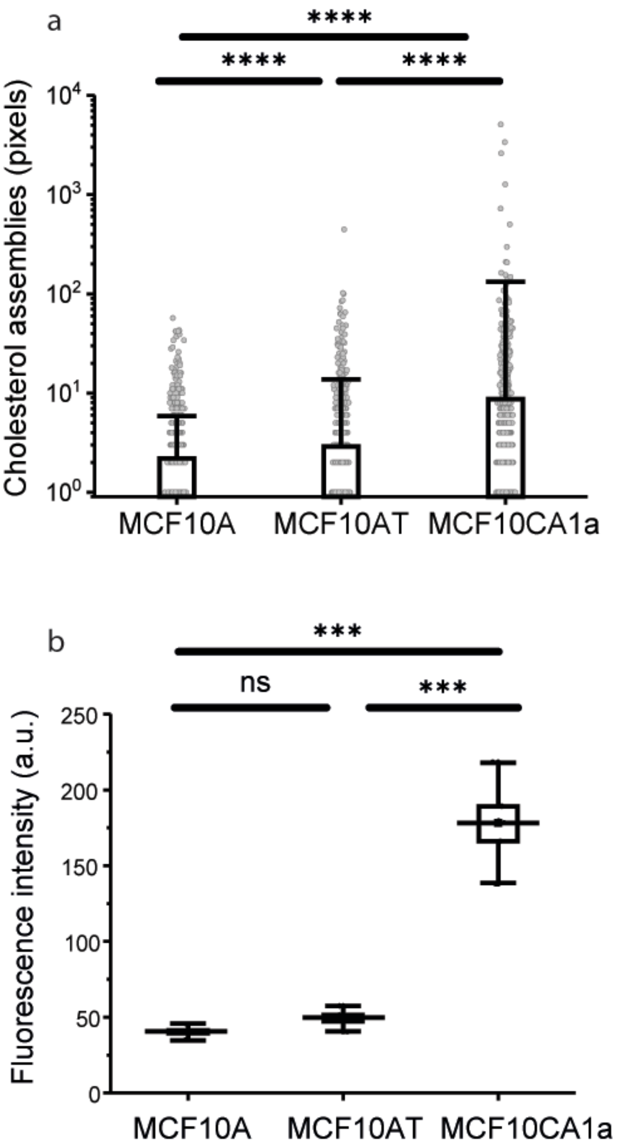

Figure S4. Quantification of the presence of plasma membrane cholesterol on the surface of living cells. (**a**) Bar plot of cholesterol assemblies size (in pixels) for MCF10 cells. FD-AFM adhesion maps of MCF10A (healthy), MCF10AT (premalignant) and MCF10CA1 (malignant) cells were analyzed and heterogeneities displaying continuous white (adhesive) pixels were marked as cholesterol assemblies. The number of pixels comprised in each cholesterol assembly was calculated. The size of detected cholesterol assemblies increases with the malignant level. (**b**) Mean fluorescence intensity values measured on the free side of mCherry-labeled MCF10 cells imaged with confocal microscopy. In panel **a**, data points correspond to individual cholesterol assemblies measured on n=5 cells/condition. Bar plot depicts mean values for each condition. Data points in panel **b** correspond to mean values measured on a single cell with n=6 cells/condition. Box plot depicts 25th -75th percentiles. Horizontal lines show mean values and error bars indicate s.d. Distributions in panel a were evaluated applying the Mann–Whitney U test. For panels b, distributions were evaluated using one-way ANOVA followed by post-hoc Tukey's HSD tests. ***$p<0.005$, ****$p<0.001$ and n.s. non-significant. All data is representative for at least 3 independent experiments.
